# Penta­aqua­(1*H*-benzimidazole-5,6-di­carboxyl­ato-κ*N* ^3^)cobalt(II) penta­hydrate

**DOI:** 10.1107/S1600536809019904

**Published:** 2009-05-29

**Authors:** Wen-Dong Song, Hao Wang, Shi-Jie Li, Pei-Wen Qin, Shi-Wei Hu

**Affiliations:** a College of Science, Guang Dong Ocean University, Zhanjiang 524088, People’s Republic of China

## Abstract

In the title mononuclear complex, [Co(C_9_H_4_N_2_O_4_)(H_2_O)_5_]·5H_2_O, the Co^II^ atom exhibits a distorted octa­hedral geometry involving an N atom of a 1*H*-benzimidazole-5,6-dicarboxyl­ate ligand and five water O atoms. A supra­molecular network is generated through inter­molecular O—H⋯O hydrogen-bonding inter­actions involving the coordinated and uncoordinated water mol­ecules and the carboxyl O atoms of the organic ligand. An inter­molecular N—H⋯O hydrogen bond is also observed.

## Related literature

For the crystal structures of related compounds, see: Gao *et al.* (2008 ▶); Lo *et al.* (2007 ▶); Yao *et al.* (2008 ▶).
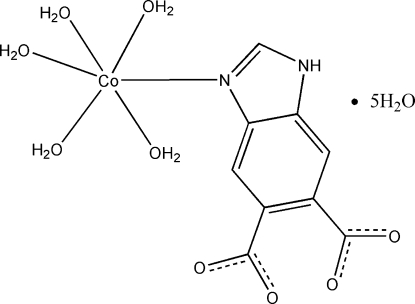


## Experimental

### 

#### Crystal data

[Co(C_9_H_4_N_2_O_4_)(H_2_O)_5_]·5H_2_O
*M* *_r_* = 443.23
Triclinic, 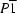

*a* = 6.8454 (14) Å
*b* = 11.480 (2) Å
*c* = 12.408 (3) Å
α = 78.02 (3)°
β = 78.57 (3)°
γ = 74.80 (3)°
*V* = 909.7 (4) Å^3^
*Z* = 2
Mo *K*α radiation
μ = 1.02 mm^−1^
*T* = 293 K
0.31 × 0.26 × 0.21 mm

#### Data collection

Rigaku/MSC Mercury CCD diffractometer
Absorption correction: multi-scan (REQAB; Jacobson, 1998 ▶) *T*_min_ = 0.744, *T*_max_ = 0.815
7307 measured reflections
3269 independent reflections
2010 reflections with *I* > 2σ(*I*)
*R*_int_ = 0.050

#### Refinement

*R*[*F* ^2^ > 2σ(*F* ^2^)] = 0.048
*wR*(*F* ^2^) = 0.148
*S* = 1.19
3269 reflections
235 parameters
30 restraints
H-atom parameters constrained
Δρ_max_ = 0.85 e Å^−3^
Δρ_min_ = −1.00 e Å^−3^

### 

Data collection: *RAPID-AUTO* (Rigaku, 1998 ▶); cell refinement: *RAPID-AUTO*; data reduction: *CrystalStructure* (Rigaku/MSC, 2002 ▶); program(s) used to solve structure: *SHELXS97* (Sheldrick, 2008 ▶); program(s) used to refine structure: *SHELXL97* (Sheldrick, 2008 ▶); molecular graphics: *ORTEPII* (Johnson, 1976 ▶); software used to prepare material for publication: *SHELXL97*.

## Supplementary Material

Crystal structure: contains datablocks I, global. DOI: 10.1107/S1600536809019904/rz2326sup1.cif

Structure factors: contains datablocks I. DOI: 10.1107/S1600536809019904/rz2326Isup2.hkl

Additional supplementary materials:  crystallographic information; 3D view; checkCIF report

## Figures and Tables

**Table 1:** Hydrogen-bond geometry (Å, °)

*D*—H⋯*A*	*D*—H	H⋯*A*	*D*⋯*A*	*D*—H⋯*A*
N2—H2⋯O10*W*^i^	0.86	1.99	2.822 (8)	162
O1*W*—H1*W*⋯O3^ii^	0.84	1.78	2.603 (7)	169
O1*W*—H2*W*⋯O6*W*^iii^	0.84	1.95	2.789 (9)	175
O2*W*—H4*W*⋯O8*W*	0.84	1.90	2.726 (9)	165
O2*W*—H3*W*⋯O4^ii^	0.84	1.78	2.614 (7)	173
O3*W*—H5*W*⋯O10*W*^iv^	0.84	1.93	2.752 (8)	167
O3*W*—H6*W*⋯O6*W*^v^	0.84	1.92	2.758 (8)	177
O4*W*—H7*W*⋯O7*W*^iii^	0.84	2.05	2.827 (7)	154
O4*W*—H8*W*⋯O1^iv^	0.84	1.96	2.801 (8)	176
O5*W*—H9*W*⋯O7*W*	0.84	1.92	2.734 (9)	162
O5*W*—H10*W*⋯O2^vi^	0.84	1.88	2.700 (7)	164
O6*W*—H12*W*⋯O1^vi^	0.84	1.98	2.812 (6)	171
O6*W*—H11*W*⋯O2*W*	0.84	2.06	2.865 (6)	161
O7*W*—H13*W*⋯O8*W*	0.84	1.89	2.721 (8)	168
O7*W*—H14*W*⋯O2^i^	0.84	1.91	2.737 (8)	168
O8*W*—H15*W*⋯O1*W*^vii^	0.84	2.05	2.860 (7)	163
O8*W*—H16*W*⋯O9*W*	0.84	1.88	2.699 (7)	166
O9*W*—H17*W*⋯O4^vii^	0.84	1.93	2.766 (9)	172
O9*W*—H18*W*⋯O3	0.84	1.93	2.771 (8)	175
O10*W*—H20*W*⋯O1	0.87	1.89	2.747 (7)	168
O10*W*—H19*W*⋯O2^vii^	0.87	2.54	3.191 (9)	133

Symmetry codes: (i) 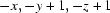; (ii) 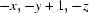; (iii) 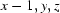; (iv) 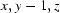; (v) 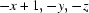; (vi) 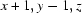; (vii) 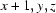.

**Fig. 1.**
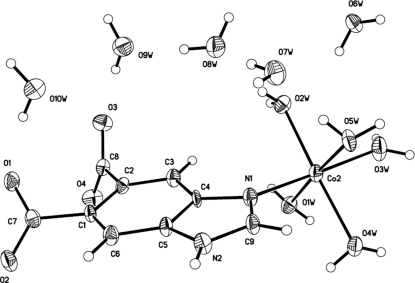
The structure of the title compound, showing the atomic numbering scheme. Non-H atoms are shown with 30% probability displacement ellipsoids.

**Fig. 2.**
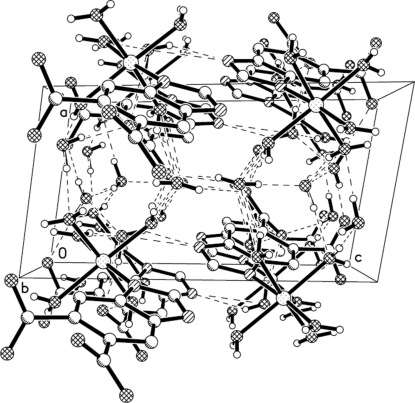
Packing diagram of the title compound viewed along the b axis. Intermolecular hydrogen bonds are shown as dashed lines.

## supplementary crystallographic information

### Comment

In the structural investigation of 1*H*-benzimidazole-5,6-dicarboxylate complexes, it has been found that the 1*H*-benzimidazole-5,6-dicarboxylic acid can function as a multidentate ligand (Gao *et al.*, 2008; Lo *et al.*, 2007; Yao *et al.*, 2008), with versatile binding and coordination modes. In this paper, we report the crystal structure of the title compound, a new cobalt(II) complex obtained by the reaction of the 1*H*-benzimidazole-5,6-dicarboxylic acid and cobalt chloride in alkaline aqueous solution.

As illustrated in Figure 1, the cobalt(II) atom is six-coordinated by one N atom from a 1*H*-benzimidazole-5,6-dicarboxylate ligand and five O atoms from five water molecules, displaying a distorted octahedral geometry. The O1/O2/C7 and O3/O4/C8 carboxylate groups are tilted with respect to the plane of the benzimidazole ring system by 36.0 (3) and 68.1 (2)°, respectively. Intermolecular O—H···O hydrogen bonding interactions (Table 1) form a three-dimensional supramolecular network involving the coordinated and uncoordinated water molecules as donors and the carboxylate O atoms of the organic ligand as acceptors (Fig. 2). An intermolecular N—H···O hydrogen bond is also observed.

### Experimental

A mixture of cobalt chloride (1 mmol), 1*H*-benzimidazole-5,6-dicarboxylic acid (1 mmol), NaOH (1.5 mmol) and H_2_O (12 ml) was placed in a 23 ml Teflon reactor, heated to 433 K for three days and then cooled to room temperature at a rate of 10 K h^-1^. The crystals obtained were washed with water and dryed in air.

### Refinement

Carbon and nitrogen bound H atoms were placed at calculated positions and were treated as riding on the parent atoms, with C—H = 0.93 Å, N—H = 0.86 Å, and with *U*_iso_(H) = 1.2 *U*_eq_(C, N). The water H atoms were located in a difference map and were refined with distance restraints of O—H = 0.84 Å, H···H = 1.39 Å and with *U*_iso_ = 1.5 *U*_eq_(O).

### Crystal data

**Table:**

[Co(C_9_H_4_N_2_O_4_)(H_2_O)_5_]·5H_2_O	*Z* = 2
*M**_r_* = 443.23	*F*(000) = 462
Triclinic, *P*1	*D*_x_ = 1.618 Mg m^−^^3^
Hall symbol: -P 1	Mo *K*α radiation, λ = 0.71073 Å
*a* = 6.8454 (14) Å	Cell parameters from 3600 reflections
*b* = 11.480 (2) Å	θ = 1.4–28°
*c* = 12.408 (3) Å	µ = 1.02 mm^−^^1^
α = 78.02 (3)°	*T* = 293 K
β = 78.57 (3)°	Block, pink
γ = 74.80 (3)°	0.31 × 0.26 × 0.21 mm
*V* = 909.7 (4) Å^3^	

### Data collection

**Table:**

Rigaku/MSC Mercury CCD diffractometer	3269 independent reflections
Radiation source: fine-focus sealed tube	2010 reflections with *I* > 2σ(*I*)
graphite	*R*_int_ = 0.050
ω scans	θ_max_ = 25.2°, θ_min_ = 3.1°
Absorption correction: multi-scan (REQAB; Jacobson, 1998)	*h* = −8→8
*T*_min_ = 0.744, *T*_max_ = 0.815	*k* = −13→13
7307 measured reflections	*l* = −13→14

### Refinement

**Table:**

Refinement on *F*^2^	Primary atom site location: structure-invariant direct methods
Least-squares matrix: full	Secondary atom site location: difference Fourier map
*R*[*F*^2^ > 2σ(*F*^2^)] = 0.048	Hydrogen site location: inferred from neighbouring sites
*wR*(*F*^2^) = 0.148	H-atom parameters constrained
*S* = 1.19	*w* = 1/[σ^2^(*F*_o_^2^) + (0.025*P*)^2^ + 3.508*P*] where *P* = (*F*_o_^2^ + 2*F*_c_^2^)/3
3269 reflections	(Δ/σ)_max_ < 0.001
235 parameters	Δρ_max_ = 0.85 e Å^−^^3^
30 restraints	Δρ_min_ = −1.00 e Å^−^^3^

### Special details

**Table:**

Geometry. All e.s.d.'s (except the e.s.d. in the dihedral angle between two l.s. planes) are estimated using the full covariance matrix. The cell e.s.d.'s are taken into account individually in the estimation of e.s.d.'s in distances, angles and torsion angles; correlations between e.s.d.'s in cell parameters are only used when they are defined by crystal symmetry. An approximate (isotropic) treatment of cell e.s.d.'s is used for estimating e.s.d.'s involving l.s. planes.
Refinement. Refinement of *F*^2^ against ALL reflections. The weighted *R*-factor *wR* and goodness of fit *S* are based on *F*^2^, conventional *R*-factors *R* are based on *F*, with *F* set to zero for negative *F*^2^. The threshold expression of *F*^2^ > σ(*F*^2^) is used only for calculating *R*-factors(gt) *etc*. and is not relevant to the choice of reflections for refinement. *R*-factors based on *F*^2^ are statistically about twice as large as those based on *F*, and *R*- factors based on ALL data will be even larger.

### Fractional atomic coordinates and isotropic or equivalent isotropic displacement parameters (Å^2^)

**Table:**

	*x*	*y*	*z*	*U*_iso_*/*U*_eq_	
Co2	0.10067 (16)	0.09663 (9)	0.24088 (8)	0.0301 (3)	
O1	−0.1942 (8)	0.8137 (4)	0.2444 (4)	0.0386 (13)	
O2	−0.4507 (8)	0.7825 (5)	0.3797 (4)	0.0417 (13)	
O3	0.0523 (8)	0.6536 (5)	0.0471 (4)	0.0420 (14)	
O4	−0.2859 (8)	0.6922 (5)	0.0637 (4)	0.0431 (14)	
N1	−0.0099 (9)	0.2334 (5)	0.3409 (5)	0.0292 (14)	
N2	−0.1506 (9)	0.3081 (5)	0.4971 (5)	0.0351 (15)	
H2	−0.1939	0.3096	0.5668	0.042*	
C1	−0.2252 (10)	0.6102 (6)	0.3117 (6)	0.0272 (15)	
C2	−0.1306 (10)	0.5624 (6)	0.2126 (6)	0.0272 (16)	
C3	−0.0533 (11)	0.4390 (6)	0.2134 (6)	0.0292 (16)	
H3	0.0095	0.4084	0.1481	0.035*	
C4	−0.0722 (10)	0.3611 (6)	0.3154 (6)	0.0259 (15)	
C5	−0.1612 (11)	0.4083 (6)	0.4130 (5)	0.0257 (15)	
C6	−0.2406 (11)	0.5323 (6)	0.4127 (6)	0.0328 (17)	
H6	−0.3025	0.5623	0.4784	0.039*	
C7	−0.2974 (11)	0.7460 (7)	0.3101 (6)	0.0323 (17)	
C8	−0.1215 (11)	0.6451 (6)	0.0995 (6)	0.0311 (17)	
C9	−0.0613 (11)	0.2089 (6)	0.4507 (6)	0.0320 (17)	
H9	−0.0373	0.1301	0.4913	0.038*	
O1W	−0.1050 (7)	0.1798 (4)	0.1266 (4)	0.0365 (12)	
H1W	−0.0713	0.2310	0.0718	0.055*	
H2W	−0.1628	0.1323	0.1082	0.055*	
O2W	0.3202 (7)	0.1855 (4)	0.1370 (4)	0.0351 (12)	
H4W	0.3630	0.2256	0.1731	0.053*	
H3W	0.2982	0.2251	0.0739	0.053*	
O3W	0.2255 (9)	−0.0454 (5)	0.1511 (5)	0.0526 (16)	
H5W	0.2351	−0.1196	0.1787	0.079*	
H6W	0.2442	−0.0342	0.0811	0.079*	
O4W	−0.1232 (8)	0.0001 (4)	0.3351 (4)	0.0370 (12)	
H7W	−0.2302	0.0564	0.3368	0.056*	
H8W	−0.1389	−0.0575	0.3079	0.056*	
O5W	0.2965 (8)	0.0074 (4)	0.3565 (4)	0.0393 (13)	
H9W	0.3548	0.0620	0.3604	0.059*	
H10W	0.3815	−0.0593	0.3500	0.059*	
O6W	0.6987 (8)	0.0165 (5)	0.0785 (4)	0.0404 (13)	
H12W	0.7395	−0.0481	0.1221	0.061*	
H11W	0.5769	0.0507	0.1004	0.061*	
O7W	0.5026 (8)	0.1541 (5)	0.4139 (5)	0.0472 (14)	
H13W	0.5043	0.2127	0.3607	0.071*	
H14W	0.4695	0.1786	0.4757	0.071*	
O8W	0.5118 (8)	0.3188 (5)	0.2216 (5)	0.0501 (15)	
H15W	0.6299	0.2925	0.1884	0.075*	
H16W	0.4733	0.3952	0.2051	0.075*	
O9W	0.4165 (9)	0.5583 (5)	0.1328 (5)	0.0547 (16)	
H17W	0.5089	0.5965	0.1059	0.082*	
H18W	0.3098	0.5902	0.1038	0.082*	
O10W	0.2113 (8)	0.7246 (5)	0.2679 (4)	0.0452 (14)	
H20W	0.0877	0.7635	0.2566	0.068*	
H19W	0.2901	0.7741	0.2624	0.068*	

### Atomic displacement parameters (Å^2^)

**Table:**

	*U*^11^	*U*^22^	*U*^33^	*U*^12^	*U*^13^	*U*^23^
Co2	0.0367 (6)	0.0223 (5)	0.0290 (5)	−0.0043 (4)	−0.0046 (4)	−0.0024 (4)
O1	0.047 (3)	0.024 (3)	0.040 (3)	−0.007 (2)	−0.002 (3)	−0.001 (2)
O2	0.049 (3)	0.027 (3)	0.041 (3)	0.002 (3)	0.000 (3)	−0.007 (2)
O3	0.039 (3)	0.044 (3)	0.033 (3)	−0.008 (3)	−0.001 (2)	0.009 (3)
O4	0.037 (3)	0.049 (3)	0.038 (3)	−0.010 (3)	−0.014 (2)	0.011 (3)
N1	0.038 (3)	0.022 (3)	0.025 (3)	−0.003 (3)	−0.005 (3)	−0.002 (3)
N2	0.049 (4)	0.029 (3)	0.020 (3)	−0.004 (3)	0.000 (3)	0.000 (3)
C1	0.032 (4)	0.014 (3)	0.033 (4)	0.000 (3)	−0.006 (3)	−0.004 (3)
C2	0.033 (4)	0.018 (4)	0.029 (4)	−0.006 (3)	−0.011 (3)	0.004 (3)
C3	0.040 (4)	0.025 (4)	0.023 (4)	−0.006 (3)	−0.006 (3)	−0.005 (3)
C4	0.032 (4)	0.009 (3)	0.032 (4)	0.002 (3)	−0.003 (3)	−0.003 (3)
C5	0.042 (4)	0.017 (3)	0.016 (3)	−0.004 (3)	−0.004 (3)	0.000 (3)
C6	0.044 (4)	0.028 (4)	0.026 (4)	−0.008 (3)	−0.003 (3)	−0.008 (3)
C7	0.034 (4)	0.026 (4)	0.034 (4)	0.001 (3)	−0.010 (3)	−0.002 (3)
C8	0.037 (4)	0.026 (4)	0.033 (4)	−0.008 (3)	−0.008 (3)	−0.006 (3)
C9	0.042 (4)	0.018 (4)	0.030 (4)	0.000 (3)	−0.004 (3)	0.000 (3)
O1W	0.042 (3)	0.032 (3)	0.035 (3)	−0.011 (2)	−0.007 (2)	0.002 (2)
O2W	0.042 (3)	0.034 (3)	0.030 (3)	−0.013 (2)	−0.008 (2)	0.002 (2)
O3W	0.080 (4)	0.029 (3)	0.042 (3)	−0.007 (3)	0.004 (3)	−0.009 (3)
O4W	0.047 (3)	0.025 (3)	0.039 (3)	−0.009 (2)	−0.005 (2)	−0.006 (2)
O5W	0.043 (3)	0.021 (3)	0.050 (3)	0.004 (2)	−0.014 (3)	−0.006 (2)
O6W	0.038 (3)	0.034 (3)	0.043 (3)	0.002 (2)	−0.007 (2)	−0.005 (3)
O7W	0.055 (4)	0.045 (3)	0.042 (3)	−0.006 (3)	−0.007 (3)	−0.013 (3)
O8W	0.050 (3)	0.039 (3)	0.061 (4)	−0.008 (3)	−0.012 (3)	−0.006 (3)
O9W	0.044 (3)	0.045 (4)	0.070 (4)	−0.010 (3)	−0.009 (3)	0.001 (3)
O10W	0.049 (3)	0.048 (4)	0.040 (3)	−0.015 (3)	−0.010 (3)	−0.001 (3)

### Geometric parameters (Å, °)

**Table:**

Co2—O3W	2.068 (5)	C5—C6	1.384 (9)
Co2—O5W	2.082 (5)	C6—H6	0.9300
Co2—N1	2.096 (6)	C9—H9	0.9300
Co2—O1W	2.104 (5)	O1W—H1W	0.8400
Co2—O2W	2.109 (5)	O1W—H2W	0.8401
Co2—O4W	2.141 (5)	O2W—H4W	0.8400
O1—C7	1.250 (8)	O2W—H3W	0.8400
O2—C7	1.259 (9)	O3W—H5W	0.8400
O3—C8	1.255 (8)	O3W—H6W	0.8400
O4—C8	1.239 (8)	O4W—H7W	0.8401
N1—C9	1.328 (9)	O4W—H8W	0.8401
N1—C4	1.401 (8)	O5W—H9W	0.8400
N2—C9	1.330 (9)	O5W—H10W	0.8400
N2—C5	1.380 (8)	O6W—H12W	0.8400
N2—H2	0.8600	O6W—H11W	0.8400
C1—C6	1.383 (9)	O7W—H13W	0.8400
C1—C2	1.419 (10)	O7W—H14W	0.8400
C1—C7	1.503 (9)	O8W—H15W	0.8400
C2—C3	1.376 (9)	O8W—H16W	0.8400
C2—C8	1.522 (9)	O9W—H17W	0.8400
C3—C4	1.394 (9)	O9W—H18W	0.8400
C3—H3	0.9300	O10W—H20W	0.8708
C4—C5	1.392 (9)	O10W—H19W	0.8660
			
O3W—Co2—O5W	88.5 (2)	N2—C5—C4	105.4 (6)
O3W—Co2—N1	175.5 (2)	C6—C5—C4	122.0 (6)
O5W—Co2—N1	87.0 (2)	C1—C6—C5	117.9 (6)
O3W—Co2—O1W	90.5 (2)	C1—C6—H6	121.0
O5W—Co2—O1W	177.2 (2)	C5—C6—H6	121.0
N1—Co2—O1W	94.1 (2)	O1—C7—O2	124.7 (7)
O3W—Co2—O2W	86.2 (2)	O1—C7—C1	117.8 (6)
O5W—Co2—O2W	93.4 (2)	O2—C7—C1	117.3 (6)
N1—Co2—O2W	94.0 (2)	O4—C8—O3	125.3 (7)
O1W—Co2—O2W	89.15 (19)	O4—C8—C2	117.0 (6)
O3W—Co2—O4W	90.0 (2)	O3—C8—C2	117.5 (6)
O5W—Co2—O4W	89.0 (2)	N1—C9—N2	113.4 (6)
N1—Co2—O4W	90.0 (2)	N1—C9—H9	123.3
O1W—Co2—O4W	88.33 (19)	N2—C9—H9	123.3
O2W—Co2—O4W	175.4 (2)	Co2—O1W—H1W	119.1
C9—N1—C4	104.2 (6)	Co2—O1W—H2W	115.2
C9—N1—Co2	122.8 (5)	H1W—O1W—H2W	111.5
C4—N1—Co2	132.5 (4)	Co2—O2W—H4W	110.6
C9—N2—C5	107.7 (6)	Co2—O2W—H3W	120.7
C9—N2—H2	126.2	H4W—O2W—H3W	111.6
C5—N2—H2	126.2	Co2—O3W—H5W	123.9
C6—C1—C2	120.1 (6)	Co2—O3W—H6W	122.3
C6—C1—C7	119.0 (6)	H5W—O3W—H6W	112.1
C2—C1—C7	120.8 (6)	Co2—O4W—H7W	101.5
C3—C2—C1	121.6 (6)	Co2—O4W—H8W	116.2
C3—C2—C8	117.0 (6)	H7W—O4W—H8W	110.5
C1—C2—C8	121.3 (6)	Co2—O5W—H9W	102.5
C2—C3—C4	117.8 (6)	Co2—O5W—H10W	123.2
C2—C3—H3	121.1	H9W—O5W—H10W	111.2
C4—C3—H3	121.1	H12W—O6W—H11W	111.4
C5—C4—C3	120.5 (6)	H13W—O7W—H14W	111.5
C5—C4—N1	109.3 (6)	H15W—O8W—H16W	111.6
C3—C4—N1	130.2 (6)	H17W—O9W—H18W	111.6
N2—C5—C6	132.6 (6)	H20W—O10W—H19W	112.0

### Hydrogen-bond geometry (Å, °)

**Table:**

*D*—H···*A*	*D*—H	H···*A*	*D*···*A*	*D*—H···*A*
N2—H2···O10W^i^	0.86	1.99	2.822 (8)	162
O1W—H1W···O3^ii^	0.84	1.78	2.603 (7)	169
O1W—H2W···O6W^iii^	0.84	1.95	2.789 (9)	175
O2W—H4W···O8W	0.84	1.90	2.726 (9)	165
O2W—H3W···O4^ii^	0.84	1.78	2.614 (7)	173
O3W—H5W···O10W^iv^	0.84	1.93	2.752 (8)	167
O3W—H6W···O6W^v^	0.84	1.92	2.758 (8)	177
O4W—H7W···O7W^iii^	0.84	2.05	2.827 (7)	154
O4W—H8W···O1^iv^	0.84	1.96	2.801 (8)	176
O5W—H9W···O7W	0.84	1.92	2.734 (9)	162
O5W—H10W···O2^vi^	0.84	1.88	2.700 (7)	164
O6W—H12W···O1^vi^	0.84	1.98	2.812 (6)	171
O6W—H11W···O2W	0.84	2.06	2.865 (6)	161
O7W—H13W···O8W	0.84	1.89	2.721 (8)	168
O7W—H14W···O2^i^	0.84	1.91	2.737 (8)	168
O8W—H15W···O1W^vii^	0.84	2.05	2.860 (7)	163
O8W—H16W···O9W	0.84	1.88	2.699 (7)	166
O9W—H17W···O4^vii^	0.84	1.93	2.766 (9)	172
O9W—H18W···O3	0.84	1.93	2.771 (8)	175
O10W—H20W···O1	0.87	1.89	2.747 (7)	168
O10W—H19W···O2^vii^	0.87	2.54	3.191 (9)	133

Symmetry codes: (i) −*x*, −*y*+1, −*z*+1; (ii) −*x*, −*y*+1, −*z*; (iii) *x*−1, *y*, *z*; (iv) *x*, *y*−1, *z*; (v) −*x*+1, −*y*, −*z*; (vi) *x*+1, *y*−1, *z*; (vii) *x*+1, *y*, *z*.
